# Rare Trafficking CFTR Mutations Involve Distinct Cellular Retention Machineries and Require Different Rescuing Strategies

**DOI:** 10.3390/ijms23010024

**Published:** 2021-12-21

**Authors:** Sofia S. Ramalho, Iris A. L. Silva, Margarida D. Amaral, Carlos M. Farinha

**Affiliations:** BioISI—Biosystems & Integrative Sciences Institute, Faculty of Sciences, University of Lisboa, Campo Grande, C8 bdg, 1749-016 Lisboa, Portugal; ssramalho@fc.ul.pt (S.S.R.); iasilva@fc.ul.pt (I.A.L.S.); msamaral@fc.ul.pt (M.D.A.)

**Keywords:** cystic fibrosis, rare mutations, protein trafficking, intestinal organoids, revertants, personalized medicine

## Abstract

Most of the ~2100 CFTR variants so far reported are very rare and still uncharacterized regarding their cystic fibrosis (CF) disease liability. Since some may respond to currently approved modulators, characterizing their defect and response to these drugs is essential. Here we aimed characterizing the defect associated with four rare missense (likely Class II) CFTR variants and assess their rescue by corrector drugs. We produced CFBE cell lines stably expressing CFTR with W57G, R560S, H1079P and Q1100P, assessed their effect upon CFTR expression and maturation and their rescue by VX-661/VX-445 correctors. Results were validated by forskolin-induced swelling assay (FIS) using intestinal organoids from individuals bearing these variants. Finally, knock-down (KD) of genes previously shown to rescue F508del-CFTR was assessed on these mutants. Results show that all the variants preclude the production of mature CFTR, confirming them as Class II mutations. None of the variants responded to VX-661 but the combination rescued H1079P- and Q1100P-CFTR. The KD of factors that correct F508del-CFTR retention only marginally rescued R560S- and H1079P-CFTR. Overall, data evidence that Class II mutations induce distinct molecular defects that are neither rescued by the same corrector compounds nor recognized by the same cellular machinery, thus requiring personalized drug discovery initiatives.

## 1. Introduction

Cystic fibrosis (CF) is the most common lethal genetic recessive disorder among Caucasians [1], affecting 1:2500–6000 newborns, depending on the geographic region [2]. CF is caused by mutations in the CF transmembrane conductance regulator (CFTR) gene [3,4], which encodes a cAMP-regulated chloride (Cl-) and bicarbonate (HCO3-) channel expressed at the apical membrane of epithelial cells [5], that also regulates other channels and transporters [6]. CF-causing mutations disrupt CFTR traffic and function leading to abnormal ion transport and causing dehydration of the airway surface liquid [7].

Although more than 2100 variants have been reported to date in the CFTR gene [8], disease liability is only confirmed for about 360 [9]. The most common mutation—F508del—is present in 80% of individuals with CF worldwide [8,9,10]. This mutant causes CFTR misfolding leading to endoplasmic reticulum (ER) retention by the ER quality control (ERQC), premature degradation and failure to reach the plasma membrane (PM). Identification of small molecules that rescue F508del-CFTR defect resulted in the approval of three corrector drugs currently available for individuals with CF: VX-809 (lumacaftor), VX-661 (tezacaftor) or VX-445 (elexacaftor—combined with VX-661), all used in combination with VX-770 (ivacaftor), a gating potentiator [11,12,13]. Ultimately, all variants result in abnormal Cl- secretion by epithelial cells and, according to the impact in CFTR, they may result in “classical” or “atypical” forms of CF disease, associated with total absence or residual Cl- transport through CFTR, respectively [14,15].

Among such a large number of variants, many of them are very rare due to their low frequency (over 1000 CFTR mutations exist in less than five individuals with CF worldwide) and have unknown impact in terms of their molecular defect and disease liability [16]. To better tackle this high number of mutations regarding their therapeutic rescue, they have been grouped into seven classes [10] according to their functional defect and ideally to be targeted by the same therapeutic strategy—‘theratypes’ [17]. The rationale to group the mutations into theratypes is that the same therapeutic approach can rescue mutants within the same class [10]. Although this classification is helpful, it is still missing for most rare variants—for which the underlying defect is uncharacterized. As at least some of these rare variants are likely respond to approved CFTR modulators [10,18], there is an unmet need to characterize them and their responsiveness to rescuing strategies [18,19,20].

Here we focused on four rare missense variants that, due to their location within CFTR structure, are likely to impair CFTR trafficking: W57G, R560S, H1079P and Q1100P. Our aim was to characterize these variants in terms of impact upon CFTR expression and maturation, response to the approved modulators both in cellular models and, when available, patient-derived materials from individuals with CF bearing these variants. Furthermore, we also aimed to understand whether each of the mutations shared the same cellular mechanisms as F508del (also a Class II mutation). To this end, we used a siRNA-based approach targeting genes that, when knocked-down (KD), rescue F508del [21,22,23,24,25].

Altogether the data presented here illustrate how complementary in vitro and ex vivo studies can contribute to understand the defect of rare CFTR mutations and to assess their responsiveness to CFTR modulator drugs for possible translation into clinical use. Furthermore, our data show that the knowledge about CFTR folding is still insufficient to understand the defect caused by many Class II mutations.

## 2. Results

### 2.1. Impact of W57G, R560S, H1079P and Q1100P in CFTR Processing and Their Response to VX-661 and VX-445

**Figure 1:**
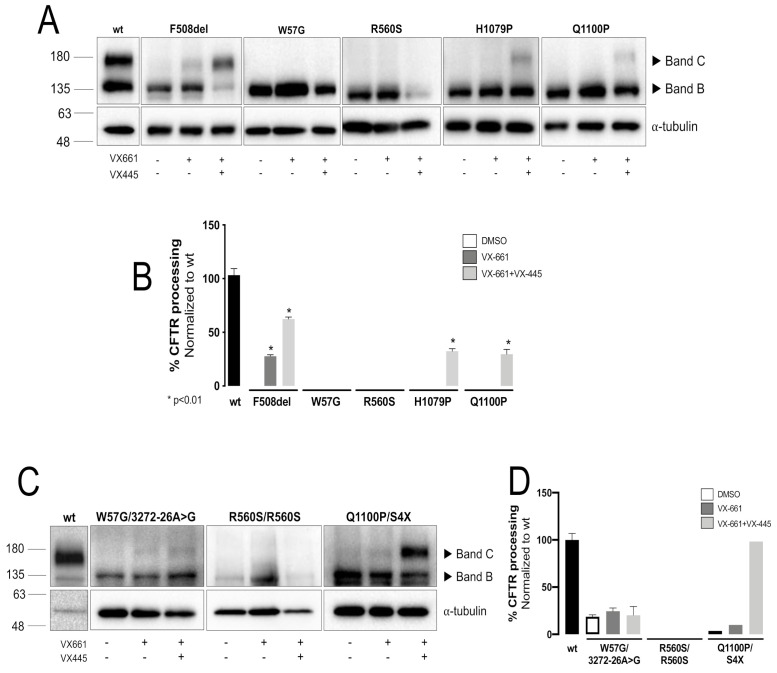
CFTR expression and effect of CFTR modulators. Representative WB analysis of CFBE cells (**A**) and organoids (**C**) expressing F508del-, W57G-, R560S-, H1079P- and Q1100P-CFTR, following incubation with DMSO 0.1% (*v*/*v*), VX-661 (5 μM) alone or in combination with VX-445 (3 μM) for 24h (*n* = 3). (**B**,**D**) For each condition densitometry was used to calculate the percentage of mature CFTR (Band C/Band B + Band C). Data were normalized to the efficiency of processing of wt-CFTR and is shown as mean ± SEM. Asterisks indicate significant difference compared with DMSO (*p*-value < 0.05, unpaired *t* test). Images were acquired using ChemiDoc XRS+ imaging system BIO-RAD and further processed by Image Lab 6.0.1 software. *, *p*-value < 0.05.

To characterize the effect of the four CFTR mutants on trafficking and response to modulators, we developed cellular models expressing each mutation individually. Cells were then incubated for 24h with 5µM VX-661/tezacaftor alone or in combination with 3 µM VX-445/elexacaftor or DMSO 0.1% (*v*/*v*), as the vehicle control. In parallel, these compounds were also tested on F508del-CFTR cells as a control. Total protein was collected, and WB was used to assess the maturation status/rescue of the four CFTR mutants (Figure 1A). Only the immature (Band B) form of CFTR was detected in control conditions for all CFTR mutants, indicating that none of them traffics through the Golgi and thus confirming them as Class II mutations. Contrarily to F508del-CFTR, which is rescued by treatment with VX-661 alone, the treatment with this corrector alone was not enough to correct the processing of any of the four mutants. However, treatment with the combination VX-661 + VX-445 rescued two of them, namely: H1079P and Q1100P at 32% and 29% of wt-CFTR, respectively. Although these levels are significant, they are lower than rescue of F508del-CFTR by the same double corrector combination, i.e., 62% of wt-CFTR (Figure 1A,B). No correction was observed for either W57G-CFTR or R560S-CFTR.

Our results were then validated by WB of intestinal organoids from individuals with CF bearing the following genotypes: CF1 [W57G/3272-26A > G], CF2 [R560S/R560S], CF3 [Q1100P/S4X]. WB analysis of intestinal organoids from CF1 showed that both mature and immature forms of CFTR were detected (Figure 1C,D) either in control cells or in cells treated with CFTR modulators. The mature CFTR form detected results from the 3272-26A > G mutation, an alternative splicing mutation from which some normal transcript still results being responsible for the synthesis of a small amount of wt-CFTR protein [26,27]. The lack of response of W57G is evidenced by the absence of any difference following incubation with modulators. CF2 WB data confirm previous results that R560S fail to produce mature CFTR and showed that not even the double corrector combination is able to elicit any correction (Figure 1C). Lastly, as for CFBE Q1100P cells, WB from CF3 organoids treated with the double corrector combination showed appearance of mature CFTR protein. Although the presence of S4X has been described to allow re-initiation of CFTR translation [28,29] and thus to produce some truncated mature protein, this was not detected under control conditions, suggesting that the full-length mature form, detected following the treatment with the two correctors, results from Q1100P rescue.

### 2.2. VX-661/VX-445 Rescue H1079P- and Q1100P-CFTR Function Both in CFBE Cells and Intestinal Organoids

**Figure 2:**
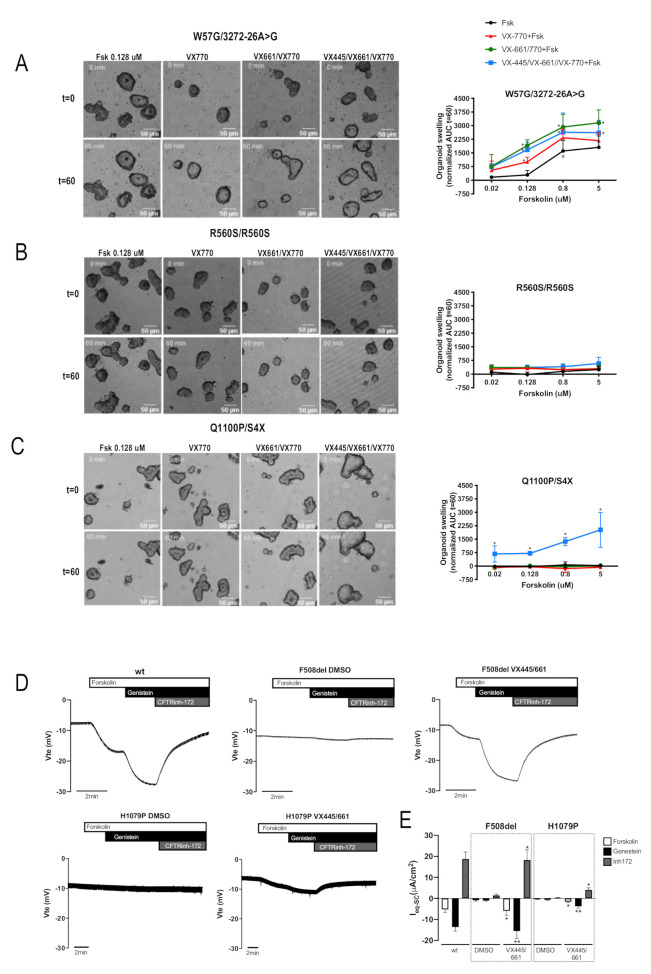
Analysis of W57G, R560S, H1079P and Q1100P effect in CFTR activity. FIS assay showing the CFTR response to treatment with VX-661 and VX-445 in intestinal organoids bearing the mutations W57G (**A**), R560S (**B**) and Q1100P (**C**). Asterisks indicate significant difference compared with Fsk (*p*-value < 0.05, ANOVA). Cardinals indicate significant difference compared with 0.02 µM Fsk (*p*-value < 0.05, ANOVA). (**D**) Original Ussing chamber (open-circuit) recordings showing transepithelial voltage measurements (Vte) obtained from CFBE cells expressing wt-, F508del- and H1079P-CFTR. (**E**) Graph summarizing equivalent short-circuit currents (I_eq-sc_) after simulation with forskolin (0.128 μM) obtained in D. A low Cl^-^ Ringer solution was used at apical side to stablish a Cl^-^ gradient. Asterisks indicate significant difference compared with DMSO (* *p*-value < 0.05, ** *p*-value < 0.01, unpaired *t*-test).

The effect of the four variants upon CFTR function and their response to modulators was assessed using the forskolin-induced swelling (FIS) assay in rectal organoids, as described [30], or the transepithelial transport of polarized cells using Ussing chamber assays. For the FIS assay, organoids were pre-incubated for 24h with the corrector VX-661 alone or in combination with VX-445 and stimulated acutely with forskolin (Fsk) and VX-770, as stated in the figure legend (Figure 2A–C).

Data show that CF1 organoids (W57G/3272-26A > G) evidenced some residual CFTR function which, however, was only detected only at Fsk concentrations of 0.8µM and higher (Figure 2A, black line), most likely due to the 3272-26A > G allele (see above). At 0.128 µM Fsk, potentiator VX-770 alone could already induce significant organoid swelling vs. control (Figure 2A, red vs. black line), that was further increased by pre-treatment with VX-661 or VX-661 + VX-445 (Figure 2A, brown and blue lines, respectively). For CF2 (R560S/R560S), pre-treatment with either VX-661 alone or in combination with VX-445 elicited no CFTR activity, confirming previous WB results (Figure 2B). Results for CF3 organoids (Q1100P/S4X) show significant rescue of CFTR activity in the presence of the triple combination of modulators (Figure 2C, blue vs. black line). No CFTR function could be detected either with VX-661 (Figure 2C, green line) or in the absence of the CFTR drugs (Figure 2C, black line), which is also in agreement with the previous WB results.

Since we had no access to materials from individuals bearing H1079P, we used the cell line expressing this variant in the Ussing chamber assay to evaluate the functional rescue of H1079P following treatment with VX-661 + VX-445 (Figure 2D). Activation of cAMP-dependent CFTR-mediated Cl- secretion by Fsk elicited a typical lumen negative response in CFBE expressing H1079P-CFTR pre-treated with VX-661 + VX-445 (Figure 2D, lower right panel). Whereas in control cells or in those treated with VX-661 alone no CFTR-mediated Cl- secretion could be detected. The functional correction obtained for H1079P-CFTR under the double corrector combination corresponds to ~28% of wt-CFTR function (Figure 2E) which is lower than that obtained for F508del-CFTR under the same conditions (Figure 2D, upper panels). In fact, the functional rescue of F508del-CFTR with VX-661 + VX-445 (under potentiation with Fsk and genistein) is very close to wt-CFTR functional levels.

### 2.3. Effect of Selected Genes KD in the Rescue of CFTR Bearing Class II Mutations

**Figure 3:**
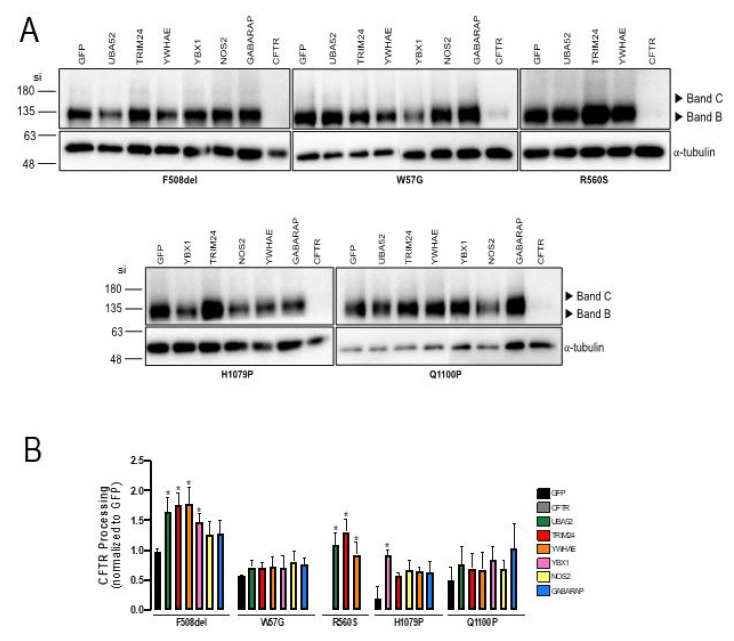
Effect of KD of selected genes in the rescue of W57G-, R560S-, H1079P- and Q1100P-CFTR folding defect. CFBE cells stably expressing W57G-, R560S-, H1079P-, Q1100P- and F508del- CFTR were transfected with siRNAs targeting 6 hit genes selected from the literature. (**A**) Representative WB analysis of CFBE cells reversely transfected with siRNAs targeting the hit genes (*n* = 4). (**B**) CFTR processing (Band C/Band B + Band C) was normalized to siRNA GFP (negative control). Asterisks indicate significant difference compared with GFP (* *p*-value < 0.05, unpaired *t* test). Images were acquired using ChemiDoc XRS+ imaging system BIO-RAD and processed using Image lab 6.0.1 software.

Several studies have shown that modulation of the cellular trafficking machinery and of specific CFTR interacting proteins promotes the rescue of F508del-CFTR [21,22,23,24,25], thus identifying putative therapeutic targets for its correction. We wondered if this approach would be successful in correcting the four rare Class II mutations under study here. For this, we selected a list of hit genes described to rescue F508del-CFTR to the PM in different studies [21,22,23,24,25] (Table S1) and performed an siRNA-based assay to assess the effect of their knock-down (KD) in cells expressing CFTR bearing these four mutations. Cells were thus subjected to reverse transfection with siRNAs against the selected genes (Table S1). Forty-eight hours after transfection, total protein was collected and used to perform WB. The siRNAs that showed a higher effect on CFTR rescue were then selected, transfected in each cell line, and run in parallel with F508del as a control (Figure 3). Effective KD of the targeted genes was confirmed by RT-PCR (Figure S1).

Transfection with these siRNAs promoted, in general, the appearance of small amounts of fully-processed F508del-CFTR (Figure 3A,B). Among the 4 rare mutants, we were able to detect a significant increase in mature CFTR only in the case of R560S (3 genes) and H1079P (1 gene), although in reduced amounts comparing to F508del-CFTR (Figure 3A). When the genes *UBA52*, *TRIM24* and *YWHAE* were KD, a small but significant amount of Band C was detected in cells expressing R560S-CFTR. Significant rescue was also detected for H1079P-CFTR under KD of *YBX1*. For W57G and Q1100P, no significant rescue was detected for any siRNA. These results suggest that, irrespectively of their ability to be rescued by modulator drugs, the folding defects caused by these four Class II mutations are structurally different from the one caused by F508del and/or that the recognition of such defect involves distinct players in the cell.

### 2.4. Effect of Second-Site Mutations in the Rescue of CFTR Bearing Class II Mutations

**Figure 4:**
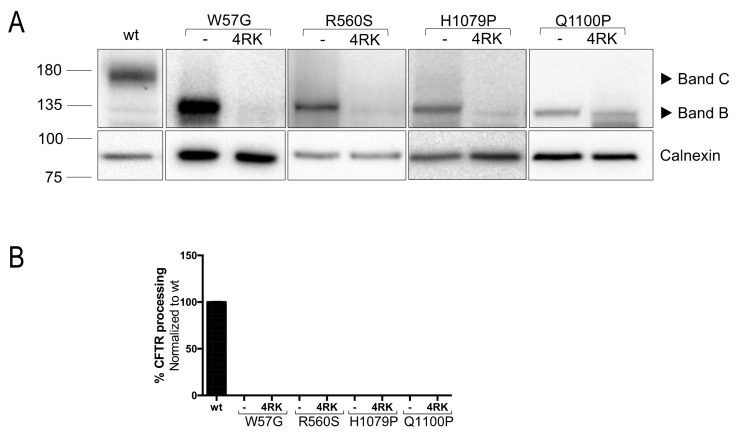
Assessment of the CFTR processing in HEK 293T cells transiently expressing W57G-, R560S-, H1079P- and Q1100P- CFTR alone or *in cis* with 4RK. (**A**) Representative WB analysis of HEK 293T cells transiently expressing W57G-, R560S-, H1079P- and Q1100P-CFTR alone and in cis with 4RK. (**B**) Densitometry was used to calculate the percentage of mature CFTR (Band C/Band B + Band C). Images were acquired using ChemiDoc XRS+ imaging system BIO-RAD and processed using Image lab 6.0.1 software.

Finally, we assessed the ability of second-site mutations (revertants) to rescue the trafficking defect caused by the four mutations under study. Although it is not entirely elucidated yet how misfolded proteins are distinguished by the ER-quality control (ERQC) from the normal proteins for either ER export or degradation, one of the known mechanisms involves the exposure of multiple arginine-framed tripeptides (AFTs) [30]. Abrogation of AFTs (by replacing selected arginine residues with lysine ones) allows mutant proteins to escape the ERQC and reach the cell surface [30]. It has been demonstrated that F508del-CFTR can be rescued by introducing the four mutations R29K, R516K, R555K, and R766K at its 4 AFTs (termed 4RK), without a correction of its folding defect [31]. To understand whether the four Class II CFTR mutants (W57G, R560S, H1079P and Q1100P) could be rescued through this approach, constructs bearing each of the mutants in cis with 4RK were developed (see Methods). HEK 293T cells were transiently transfected with these plasmids, and WB was performed (Figure 4A,B).

Unlike F508del, that was shown by us and others to be partially rescued when in cis with 4RK [24,31], none of the mutations was rescued by the presence of the 4RK in cis. These results suggest that the structural impact of these mutations in CFTR is distinct from that caused by F508del—as the alterations in the AFT motifs do not allow ER escape.

## 3. Discussion

The high number of variants described in the *CFTR* gene makes it difficult to predict disease severity for individuals bearing rare mutations and impairs their access to innovative therapies that rescue the underlying basic defect. Here, we characterized four rare CFTR variants in terms of their impact in CFTR expression and function and their response to modulators, particularly to the newly approved highly effective triple combination therapy—Tezacaftor/Elexacaftor/Ivafactor—both in cellular models constitutively expressing four CFTR mutants and in patient-derived materials (intestinal organoids). We also provide some clues on the mechanisms of disease behind these mutations by performing a siRNA-based study to find genes/protein targets possibly rescuing these mutants.

The four variants analyzed (W57G, R560S, H1079P and Q1100P) share the fact that they are extremely rare—two of them (H1079P and Q1100P) are not even listed in the CFTR2 database.

W57G is located in the lasso motif which is known to interact with the membrane trafficking machinery [32]. Mutations in this region are described to cause intracellular retention or abnormal gating [33]. W57G is described in CFTR2 [9] as CF-causing when combined with another CF-causing mutation. W57G mutation exists in a reduced number of individuals with CF (10 in total reported in CFTR2, 7 in heterozygosity with F508del). It is associated with severe CF phenotype, causing pancreatic insufficiency (PI) when combined with another PI-causing mutation. In the Cystic Fibrosis Mutation Database [8], W57G was initially described in an Italian individual, in compound heterozygosity with R352Q. Functional data in CFBE Flp-In cells describe W57G as damaging, having 1% of wt-CFTR function [34]. CF1, described in this study, is the first individual bearing W57G in compound heterozygosity with 3272-26A > G.

R560S is a rare Class II mutation that we previously characterized and determined its lack of response to either lumacaftor or tezacaftor [35]. This mutation is localized in NBD1, close to the signature motif. There are six individuals with CF described in CFTR2 as bearing R560S and it is associated with a severe CF phenotype and with PI when in combination with another PI-causing mutation. We previously analyzed materials from a homozygous individual for R560S (CF2 in this study) confirming that it is a Class II mutation, causing the total abrogation of mature CFTR and complete absence of CFTR function [35].

H1079P is a very rare mutation not described in CFTR2 and only identified in two sister siblings [8] both pancreatic insufficient (PI), and heterozygous for W1282X [8]. H1079P is located in the proximity of the intracellular loop 4 (ICL4), which interacts with NBD1 [33]. Mutations in this region, such as H1054D and L1077P, have been described to disturb the ICL4 structure, leading to processing and gating defects [36].

Q1100P is a very rare mutation, not reported in CFTR2, located in the transmembrane helix 11 (TM11) that forms a hydrophobic pocket with ICL4 where the side chain of F508 is inserted [33]. Q1100P was originally found in a Spanish individual, carrying F508del in the other allele, who is PI and presents severe lung disease [8]. Here we describe CF3, an individual with CF bearing Q1100P in heterozygosity with S4X, also PI.

We first characterized these mutants both in cellular models and in intestinal organoids by determining the levels of immature and mature CFTR by WB in CFBE cells. Data showed that all four mutations affect CFTR protein processing, totally abrogating the production of the mature form (Figure 1), thus placing these mutations in Class II, as F508del. The functional classification prompted us to test whether the approved corrector drugs were able to rescue CFTR mutants. Treatment with VX-661 (lumacaftor) alone was not able to rescue the processing defect caused by any of the mutations.

Treatment with the double corrector combination VX-661 + VX-445 lead to the appearance of mature CFTR in CFBE cells expressing H1079P and Q1100P (Figure 1), to 29% and 32% of wt-CFTR levels for H1079P- and Q1100P-CFTR, respectively. No mature CFTR could be detected for W57G- and R560S-CFTR expressing cells after treatment with the two correctors combination. Biochemical analysis in intestinal organoids from individuals bearing W57G, R560S and Q1100P confirmed the results obtained in CFBE cells.

We then assessed the functional defect associated with these mutations and again their response to modulators. Organoids from CF1 [W57G/3272-26A > G] showed residual CFTR activity at 0.8uM of Fsk, which is most likely due to the presence of some wt-CFTR protein resulting from the 3272-26A > G allele. In fact, we have shown previously that individuals with CF bearing this mutation express low levels of full-length CFTR mRNA and this leads to the appearance of some wt-CFTR at the plasma membrane [27]. Individuals with the genotype 3272-26A > G/F508del have around 5% of normal mRNA and this leads to an attenuated respiratory phenotype [37]. Results from the FIS assay on organoids show some basal CFTR activity and also upon treatment with modulators, which again is probably due to the 3272-26A > G mutation. In fact, this is one of the CFTR mutations eligible for tezacaftor/ivacaftor treatment [38]. No swelling was observed for organoids from CF2 (homozygous for R560S) confirming the biochemical data. Regarding CF3 [Q1100P/S4X], we could only detect significant CFTR activity for organoids co-treated with VX-661 + VX-445, thus confirming the response of Q1100P to modulators observed in the biochemical results. Although S4X could lead to some re-initiation of translation, we discarded this hypothesis as no truncated protein was detected. Being S4X a stop mutation that causes severe disease (according to CFTR2) and thus less likely to respond to correctors and/or potentiators, the observed response can likely be attributed to Q1100P.

For H1079P, due to the unavailability of materials from individuals bearing this mutation, Ussing chamber measurements were performed on cell lines. A significant increase in CFTR function was observed for CFBE cells expressing H1079P-CFTR treated with VX-661 + VX-445. The functional rescue observed corresponded to about 30% of wt-CFTR and it will likely translate into clinical benefit. Considering that the two only individuals described with this mutation are compound heterozygous for W1282X, it is even more likely that they will get benefit VX-661 + VX-445 + VX-770 because, although being a nonsense mutation, its location closer to the C-terminus of CFTR and may allow the existence of some truncated and functional protein [39]. However, the rescue observed for H1079P-CFTR was lower than that in cells expressing F508del-CFTR for the same drug combination, for which correction brings it to almost wt-CFTR levels. The fact that this functional correction for F508del is higher than the one obtained for processing may result from the possible dual role of VX-445/elexacaftor as both a corrector and a potentiator [40].

Our results reinforce the knowledge that not all mutations within the same class respond to the same modulators. The mechanism of action for current correctors is still poorly understood but the fact that H1079P- and Q1100P-CFTR respond to VX-445 (in combination with VX-661) suggests that the compound may bind to CFTR bearing these mutations but not to the abnormal structure elicited by W57G or R560S.

H1079P is located in ICL4, the interaction of which with NBD1 is defective in F508del-CFTR [41] being corrected, at least partially, by VX-809 [42]. A similar mechanism of action is probably occurring for VX-661, given its structural similarity to VX-809. Possibly the pocket created by the change of a Histidine to a Proline is different from that created by the deletion of Phe-508—thus explaining why VX-661 does not rescue H1079P-CFTR. However, the interdomain contacts may still create the putative NBD1 binding site of VX-445, for which the His-620 residue seems to be critical [43]. A similar rationale can be used for Q1100P, which is located in the middle of TM11, possibly influencing structure of ICL4, located in its vicinity, and thus also the NBD1-ICL4 interaction. Considering the limited knowledge regarding the binding site for correctors, the efficacy of each drug seems to be greatly dependent on the available “pockets” in CFTR structure, which are mutation-specific.

To further examine the cellular mechanisms underlying the defect caused by these four mutations, we then explored the ability to rescue them by silencing gene targets that can correct F508del-CFTR trafficking. We confirmed that knocking-down *UBA52*, *TRIM24*, *YWHAE* and *YBX1* rescue F508del-CFTR. However, we could only detect rescue of R560S- and H1079P-CFTR by some of these siRNAs. Knock-down of *UBA52*, *TRIM24* and *YWHAE* lead to the appearance of a faint Band C in cells expressing R560S-CFTR and *YBX1* KD led to small (although significant) rescue of H1079P-CFTR.

UBA52 (Ubiquitin-60S ribosomal protein L40) encodes a fusion of ubiquitin with the 60S ribosomal protein L40, which is essential for the translation of a subset of cellular transcripts. Recent studies have shown that regulatory 40S ribosomal ubiquitination is a critical phase of translational control [44]. The effect upon R560S-CFTR may be related to a potential flexibility in translation that allows some mutant CFTR to escape ERQC and reach PM, an effect that is likely to be more pronounced for a NBD1 mutation that seems to be quite difficult to correct [35].

TRIM24 (Transcription intermediary factor 1-alpha) is a transcriptional coactivator that also functions as an E3-ubiquitin ligase. The mechanism by which the KD of *TRIM24* leads to the rescue of R560S-CFTR to PM may involve the inhibition of its activity as E3-ubiquitin ligase activity allowing some of the mutant CFTR protein, by accumulating, is able to escape the ER [45].

YWHAE (14-3-3 epsilon) is part of the 14-3-3 family, which includes proteins that bind to phosphorylated substrates, affecting their enzymatic activity, cellular localization, or degradation process. Previous interactome data from our group has shown that 14-3-3ε has a higher affinity for F508del- than for wt-CFTR [24] which may be explained by the exposure of ER retention signals (AFTs). This suggests that 14-3-3ε may recognize these signals and possibly block the trafficking of misfolded proteins. The rescue of R560S-CFTR by YWHAE suggests that the misfolded CFTR recognition by 14-3-3ε may not rely only on AFTs, considering that R560S-CFTR is not rescued by the abrogation of the AFTS. KD of this gene may thus act through an alternative (AFT-independent) mechanism that allows R560S-CFTR to escape ERQC and reach the PM.

YBX1 (Y-box binding protein 1) is a DNA- and RNA-binding protein involved in processes, such as translational repression, RNA stabilization and mRNA splicing. YBX1 contributes to the regulation of translation by modulating the interaction between the mRNA and eukaryotic initiation factors [21,46]. The modest rescue of H1079P-CFTR after YBX1 KD may result from a dysregulation of the co-translational control network leading to CFTR processing.

Finally, we assessed the effect of 4RK revertant in the rescue of CFTR mutants [30]. However, none of the mutants was rescued by the abrogation of the AFTs. This suggests that, although rescue through AFT abrogation is caused by a failure of the ERQC to recognize the exposed retention motifs, the changes caused by these four mutations do not seem to lead to a similar exposure of these motifs, reinforcing the idea that each mutation causes very specific defects and that a correlation between global defect/class, response to available modulators and retention mechanism does not seem to occur. In fact, the observed differences in the effect of target KD upon different mutants and the lack of effect of the 4RK revertant suggest that the rescue mechanisms are strongly connected with the specific, almost unique, molecular defect caused by each one of them.

Our findings confirm that W57G, R560S, H1079P and Q1100P are Class II mutations and that the domains in which they are located are critical for CFTR folding. Although sharing the same cellular phenotype (absence of mature form) with F508del-CFTR, these mutations induce distinct molecular defects that cannot be rescued by the same corrector compounds and/or are recognized by different cellular components, thus requiring personalized drug discovery initiatives.

## 4. Materials and Methods

### 4.1. Chemicals and Compounds

All chemicals were of analytical grade. DMSO and Forskolin were from Sigma-Aldrich (St. Louis, MO, USA), VX-661 and VX-770 were from Selleckchem (Houston, TX, USA) and VX-445 was from MedChemExpress (Monmouth Junction, NJ, USA). VX-661, VX-445, VX-770 and Fsk were dissolved in DMSO. Unless otherwise stated (see legends of figures), the incubation time was 24 h and the concentrations used were (μM): 5 VX-661, 3 VX-445 and 3 VX-770 which are the standard and are in agreement with previous usage [12,47].

### 4.2. CF Subjects and Ethical Approval

Rectal biopsies were obtained from three individuals with CF upon ethical approval by the hospital’s ethical committee and informed consent, as detailed below:

CF1—A male individual with the genotype W57G/3272-26A > G, with sweat chloride of 89 mmol/L, normal body mass index (BMI), pancreatic sufficient, FEV1 predicted of 93%, bronchiectasis and colonization with *P. aeruginosa* and *S. aureus*.

CF2—A male individual homozygous for R560S, previously described [35], with sweat chloride of 100mmol/L, pancreatic insufficient, and malabsorption.

CF3—A female individual with the genotype Q1100P/S4X, with sweat chloride of 112 mmol/L, normal BMI and pancreatic insufficient.

### 4.3. Intestinal Organoids Culturing and Forskolin-Induced Swelling (FIS) Assay

Crypt isolation from rectal biopsies, organoid culturing and FIS were performed as described previously [29,48,49]. FIS quantification was performed using Cell Profiler and the area under the curve (AUC; t = 60; baseline = 100%) was calculated using GraphPad Prism 7.0. ANOVA tests with *p*-value ≤ 0.05 considered as significant.

### 4.4. Cell Lines

Cell lines were generated by lentiviral transduction of the CFBE41o- cell line [50] after site-directed mutagenesis and cloning of W57G-, R560S-, H1079P- and Q1100P-CFTR cDNAs into lentiviral plasmids as previously described [35].

### 4.5. Western Blot (WB) Analysis

For CFTR protein detection, intestinal organoids and CFBE cells were lysed in Laemmli buffer supplemented with complete protease inhibitor tablets (Roche, Basel, Switzerland). Lysates were analyzed by SDS-PAGE and transferred to a PVDF membrane (Millipore, Burlington, MA, USA). The primary antibody was the anti-CFTR monoclonal antibody (mAb) 596 (CFF) at 1:3000 dilution and the secondary mAb was horseradish peroxidase-labelled anti-mouse at 1:3000 (BioRad, Hercules, CA, USA). Alpha tubulin was detected by anti-alpha tubulin antibody (1:10,000) (Sigma, Darmstadt, Germany) as a loading control. Images were acquired using ChemiDoc XRS+ imaging system BioRad and analyzed with the Image lab 4.0 software.

### 4.6. Preparation of siRNA Coated Multi-Well Plates

Multi-well plates (BD Falcon, NY, USA) were coated with customized siRNAs (siGENOME, Dharmacon) for solid-phase reverse transfection adapted from previously reported protocols [51,52]. Briefly, an aqueous 0.2% (*w*/*v*) gelatin solution was prepared and filtered with a 0.45 μM pore size filter and a 0.4 M sucrose solution was prepared in Opti-MEM. Then, a transfection mix was prepared by mixing 277 μL of the sucrose/Opti-MEM solution, 81 μL of Dharmafect1 and 242 μL doubly distilled water. This transfection mix was distributed into a 96-conic well plate (6 μL/well, “Plate A”). Gelatin solution was distributed into another 96-conic well plate (96 μL/well, “Plate B”). Then, 2.5 μL of a 2 μM siRNA solution and 3.5μL of the transfection mix (“Plate A”) were incubated in each well of a 96 well plate (“Plate C”). After a 20 min incubation, 3.5 μL of the gelatin solution (“Plate B”) were added to Plate C. A total of 8.5 μL of the contents of each well in “Plate C” were diluted fifty-fold in a 96 deep well plate using doubly distilled water. Finally, 50 μL of each well were transferred to a 96-well plate, lyophilized, and stored in an anhydrous atmosphere before cell seeding. siRNAs targeting GFP and Luciferase were used as negative control. siRNA targeting CFTR was used a positive control. CFBE cells were grown to confluence and split to 50% confluency. Twenty-four hours later, cells were trypsinized to antibiotic-free medium and seeded in siRNA coated 96-well plates (20 × 103 cells/well). CFTR protein expression was assessed by WB 48 h post-transfection.

### 4.7. Micro-Ussing Chamber Recordings

Transepithelial electrical resistance (TEER) of the cells growing on Snapwell inserts was measured with the Chopstick Electrode (STX2 from WPI^®^). Monolayers with resistance values above 600 Ω.cm^2^ were mounted in modified micro-Ussing chambers. Recordings were performed as previously described [53,54], but using 0.128 μM of the cAMP agonist Fsk.

### 4.8. RT-PCR

RNA was isolated from CFBE cells using the NZY Total RNA Isolation kit (Nzytech, MB13402) according to manufacturer’s instructions. cDNA was generated from 1 μg mRNA using NZY M-MuLV Reverse Transcriptase (Nzytech, MB08301). RT-PCR was performed with primers specific for the following genes: *UBA52, TRIM24*, *YWHAE*, *YBX1*, *NOS2*, *GABARAP*. Products were analyzed by agarose gel electrophoresis.

### 4.9. Statistical Analyses

Data are mean values ± SEM. Statistical analyses were performed on GraphPad Prism 7.0 using two-tailed paired Student’s *t*-tests (unless otherwise stated), with *p* < 0.05 considered as significant.

## Data Availability

The data presented here are available on request from the corresponding author. The data are not publicly available due to privacy and ethical issues.

## Supplementary Materials

The following are available online at https://www.mdpi.com/article/10.3390/ijms23010024/s1.
