# Supplementary material for: Rare Trafficking CFTR Mutations Involve Distinct Cellular Retention Machineries and Require Different Rescuing Strategies
Source: Int J Mol Sci. 2021 Dec 21;23(1):24. doi: 10.3390/ijms23010024 (PMC8744605; doi:10.3390/ijms23010024)
Supplement: Supplementary file 1 [file ijms-23-00024-s001.zip › ijms-1498032-supplementary.pdf]

Supplementary Data

Table S1. List of hit genes and controls selected to be analyzed, listed according to the references from which they were retrieved.

Pankow *et al* 2015 [22]	Tomati *et al* 2018 [23]	Hutt *et al* 2018 [26]	Canato *et al* 2018 [25]	Loureiro *et al* 2019 [24]	Controls
**Gene**	**Gene**	**Gene**	**Gene**	**Gene**	**Gene**
PTBP1	*TRIM24*	*EIF3A*	*KIFC1*	*GABARAP*	*Luciferase*
YBX1	*FAU*	*CALM1*	*GET4*	*NOS2*	*CFTR*
HACD3	*UBA52*	*P4HB*	*YWHAE*	*SMURF1*	*GFP*
		PGRMC1	*FKBP4*		
		MCM7			

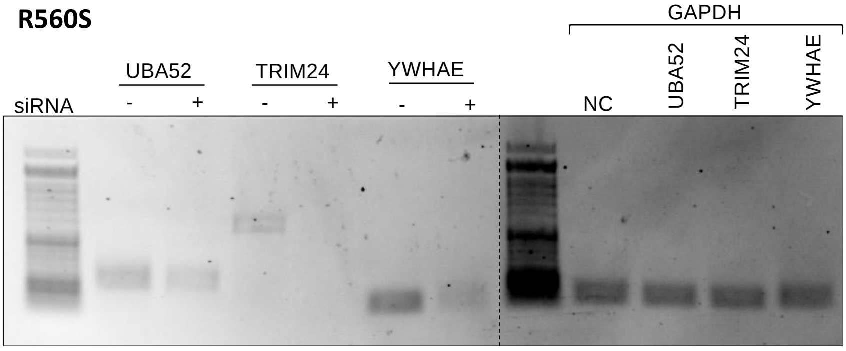

Figure S1 – Confirmation **of knock-down for selected hits by RT-PCR.** RT-PCR analysis of transcripts extracted from CFBE R560S transfected with siRNAs targeting *UBA52, TRIM24* and *YWHAE*. 1% agarose gel showing PCR products for each combination of primers specific for the following genes: *UBA52, TRIM24, YWHAE*. Bands of expected size confirming the knock-down of each of the genes 48h after transfection with siRNAs. *GAPDH* was used as a control. NC – Negative control. Expected sizes – *UBA52*: 261bp, *TRIM24*: 607bp, *YWHAE*: 90bp, *GAPDH*: 100bp.
